# Microwave Non-Destructive Inspection and Prediction of Modulus of Rupture and Modulus of Elasticity of Engineered Cementitious Composites (ECCs) Using Dual-Frequency Correlation

**DOI:** 10.3390/s17122831

**Published:** 2017-12-06

**Authors:** Kwok L. Chung, Chunwei Zhang, Yuanyuan Li, Li Sun, Mohamed Ghannam

**Affiliations:** 1 School of Civil Engineering, Qingdao University of Technology, Qingdao 266033, China; klchung@qut.edu.cn (K.L.C.); liyuanyuan@qut.edu.cn (Y.L.); 2 School of Civil Engineering, Shenyang Jianzhu University, Shenyang 110168, China; sunli@sjzu.edu.cn; 3 Structural Engineering Department, Faculty of Engineering, Mansoura University, Mansoura, Dakahlia 35516, Egypt; m.ghannam@mans.edu.eg

**Keywords:** engineered cementitious composite (ECC), electrical conductance, flexural strength, microwave non-destructive inspection (mNDI), modulus of rupture, modulus of elasticity, strength prediction, rectangular waveguide sensor, structural health monitoring, water-to-binder ratio

## Abstract

This research article presents dual-frequency correlation models for predicting the growth of elasticity and flexural strength of engineered cementitious composites (ECCs) using microwave nondestructive inspection technique. Parallel measurements of microwave properties and mechanical properties of ECC specimens were firstly undertaken in the sense of cross-disciplinary experiments. Regression models were developed via means of nonlinear regression to the measured data. The purpose of the study is: (i) to monitor the flexural strength and elasticity growth; and (ii) to predict their mature values under the influence of different initial water contents, via microwave effective conductance at early ages. It has been demonstrated that both the modulus of rupture (MOR) and modulus of elasticity (MOE) can be accurately modeled and correlated by microwave conductance using exponential functions. The moduli developed as a function of conductance whereas the regression coefficient exhibited a linear relation with water-to-binder ratio. These findings have highlighted the effectiveness of the microwave non-destructive technique in inspecting the variation of liquid phase morphology of ECCs. The dual-frequency correlation can be used for structural health monitoring, which is not only for prediction but also provides a means of verification.

## 1. Introduction

Strength monitoring of concrete at early ages is gaining more attraction as it helps in deciding the concrete readiness for service and conducting significant actions such as the removal of frame work, starting pre-stressing or even cracking control. Compressive strength, modulus of elasticity (MOE), modulus of rupture (MOR), resistivity and moisture content, to name a few, are considered as the most important parameters. They need to be inspected at early ages in order to predict the service performance of concrete, or in general, the cement-based material (CBM) in mature ages. Two common approaches are currently being used in structural health monitoring to inspect CBM mechanical properties: destructive testing (DT) and nondestructive testing (NDT). DT is done by performing destructive experiments such as compression, bending test, etc., using specimens in shapes of a cube, cylinder or beam. However, such experiments are usually undertaken at the curing ages of 7 to 28 days after casting. DT could also be performed through coring concrete from an interested part of structure, which aims to inspect the safety of built/mature structures. When considering the action and processing, DT approach is known to be material wasting, manpower- and time-consuming. On the other hand, NDT approaches offer advantages of providing information on physical and mechanical properties of in situ hardened concrete at early ages without harming structural health and wasting materials. However, there is no unique approach, from either DT or NDT, which currently satisfy the health inspection requirements. The NDT approaches do not fully substitute the DT ones but rather they are used in a complementary manner.

The physical and mechanical properties of early-age CBMs can be monitored and inspected using a number of NDT techniques. These include the rebound hammer test [1], the use of embedded sensors [2,3,4,5], the ultrasonic approaches [6,7,8,9], the acoustic methods [10], the electrical resistance or resistivity tests [11,12,13,14,15], the microwave non-destructive inspection/monitoring (mNDI) techniques [16,17,18,19,20,21,22,23,24,25]. Recently using electromagnetic or microwave approaches as a NDI method has been gaining more attention especially for concrete structures [24,25,26,27,28,29,30]. Microwave signals can penetrate inside a dielectric medium (e.g., CBMs) and interact with its microstructure. The dielectric properties at microwave frequencies (e.g., 1 GHz to 10 GHz) change with time as a result of chemical reaction between water and cement, which render a strong interaction with the porosity and saturation in CBMs, thus allowing for the prediction of the durability. During the curing process, porosity is certainly produced inside CBMs; the amount is known to relate to the amount of initial water content, which renders a correlation with mechanical properties, structural health and durability performance [31,32]. The mNDI approaches have shown tremendous potential for determining a wide range of properties of CBMs using different techniques. For example, detecting locations of reinforcing bars in concrete slabs [17]. Bois et al. [18] utilized the reflection properties of an open-ended rectangular waveguide (RWG) probe radiating into CMBs at 5 GHz (G-band) and 10 GHz (X-band) to determine the state of hydration of cement paste and concrete with different water-to-cement (*w*/*c*) ratios and different aggregate contents. Mubarak et al. [19] developed a simple robust on-site mNDI technique for determining *w*/*b* of CBMs. Whilst Peer et al. [21] used reflection and dielectric properties for investigating effects of chloride and cyclical exposure to it in mortar, Chung et al. [30] developed correlation models to predict the compressive strength of ECCs with microwave conductance. Moreover, the mNDI techniques offer advantage of inspecting the desired structure without direct contact in cases where physical access is not possible, for example, CBMs exposed to high temperatures.

Engineered cementitious composite (ECC) is considered as one of the most recent advances in concrete technology which was devised and encouraged by Li [33,34,35] along with a large number of researchers worldwide [36,37,38,39,40,41,42,43,44]. ECC is a class of fiber reinforced cementitious composites which is characterized by its high tensile strength that can reach 2 to 5 times higher than the normal concrete [35,36]. Owing to the inclusion of fibers that minimize the cracks occurring, therefore improving tensile strength and flexural strength. The use of ECC can improve the service life of structures due to its ability to sustain harsh environmental conditions, thus automatically leads to reducing maintenance and repair costs [36]. Chung et al. [37] show that ECC has more ductility compared to mortar specimens of the same mix proportion without adding PVA fibers. However, other than the known effect of water-to-binder ratio which affects the strength gain during hydration [38], the distribution of fiber orientation in ECC matrix also pays an important role on its mechanical properties and performance [39,40,41].

Due to the increase demand of using ECC in composite structures it becomes very important to study different methods of NDT that can be used for structural health monitoring [45]. MOE and MOR are the two impotant mechanical properities that affect the structural behaviour of not just CBMs [46,47,48], but also common structural materials like flakeboard [49] and woods [50]. Nevertheless, to the best knowledge of the authors, there is no research on the mNDI methods in monitoring ECC concrete, in particular, the MOE and MOR, and no exploration into their relationship. This has therefore motivated us to undertake the current research. The objective of this research is to experimentally study the growth of MOR and MOE of ECCs with tightly controlled *w*/*b* ratios (0.20, 0.255, 0.30) via means of the microwave nondestructive technique. The ultimate goal is to develop dual-frequency correlation model to inspect and predict the MOR and MOE of ECCs using microwave properties.

**Figure 1:**
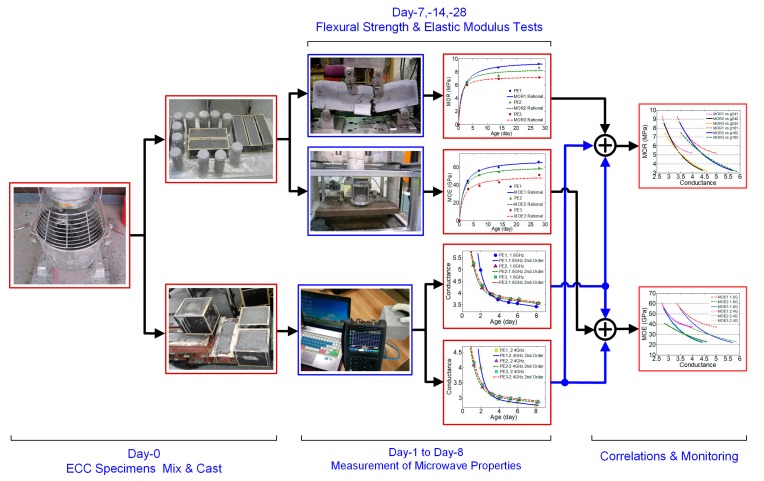
A big figure describing the logical flow of parallel experiments and the aimed dual-frequency correlations.

In this study, the authors first attempted to undertake the ECC specimens’ fabrications and then conducted the cross-discipline experimental works in parallel. Nonlinear regression was applied and analyzed to the measurement data for the MOE and MOR as well as the microwave conductance at dual frequency. Figure 1 illustrates the progression of work from day-0 mixing, casting, parallel experimental tests in accordance with standards in various days till 28 days, regression modeling and correlations established using dual-frequency approach. The achieved correction models indicate that both the MOR and MOE of ECC specimens developed as a function of conductance at 1.8 GHz and 2.4 GHz, which can be used for structural health monitoring, not just for prediction but also to verify the mechanical properties of ECC. This is the key advantage of using dual-frequency correlations.

## 2. Parallel Experimental Programs

### 2.1. ECC Mixes Design

For the purpose of getting correlations between microwave properties and mechanical properties of ECC, a set of ECC specimens of different mix proportions were made. The ECC matrix materials used were composed of cement (Type-GP cement conforms to AS3972-1997), fine silica sand (supplied by Sibelco Australia, Sydney, Australia), ASTM class-F fly ash, polyvinyl alcohol (PVA) fibers, water and high range water reducer (HRWR). A maximum grain size of 250 μm and a mean grain size of 110 μm of silica sand was used. The mechanical and physical properties of the unoiled PVA fiber is shown in Table 1, whereas the chemical compositions by percentage weight of other raw materials are listed in Table 2. The values of chemical compositions were analyzed by using the multi-purpose scanning electron microscope (SEM, JSM6510LV, JEOL Ltd., Tokyo, Japan). In this study, a standard mix design with a water-to-binder (*w*/*b*) ratio of 0.255 according to the initiative proposed by Li et al. [33,34,35] was adopted. In addition, two more ECC mixes with slightly decreased (*w*/*b* = 0.20) and increased (*w*/*b* = 0.30) initial water contents were cast in order to investigate the effects of small variation in *w*/*b* ratio to the microwave [30] and mechanical properties. The values of all mix proportions of ECC composites are summarized in Table 3, where the proportions of PVA fiber at a standard volume fraction of 2% (26 kg/m^3^) remain constant.

With the aim of getting good workability of the ECC mixture, a 40 L Hobart planar mixer was used in the experimental program. As known to all, the raw materials mixing sequence and the processing details have a great influence on the homogeneity of ECC matrix. Hence a new mixing sequence [41] was used, where the mix of solid materials with water was divided into two steps and the addition of fibers was added in several steps. Moreover, appropriate adjustment in the amount of HRWR in each mixture was performed to obtain consistent rheological properties for a better fiber distribution and workability. Then the mixture was cast into various sizes of molds and then demolded after 24 h of natural curing. All specimens, except those used for mNDI, were cured and stored in water tanks and had surface moisture removed prior to the mechanical tests. All required standard sizes and the number of ECC specimens used per type are listed in Table 4.

### 2.2. Microwave Near-Field Test

**Figure 2:**
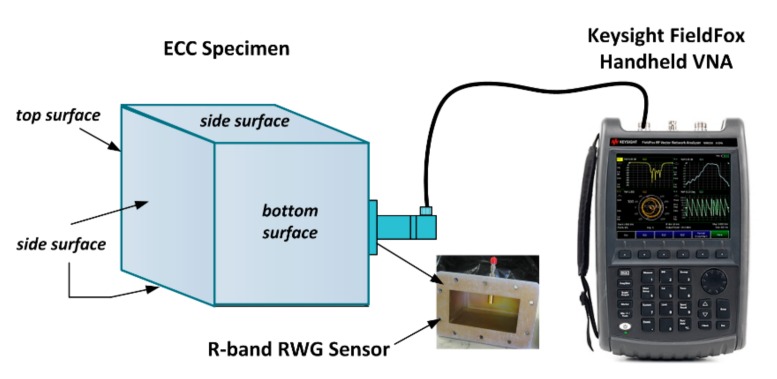
Schematic of nondestructive microwave measurement setup.

Microwave nondestructive inspection and evaluation technique has shown a great alternative inspection method to test and inspect various properties of cement-based materials [16,17,18,19,20]. However, almost all of these experiments were conducted inside laboratories, which restricted the convenient usages of microwave near-field technique. In this study, the authors attempted to use a handheld vector network analyzer (VNA) by which the nondestructive test can be undertaken in the construction site instead of a microwave laboratory. Figure 2 shows the schematic of microwave measurement setup where the FieldFox VNA N9923A (Keysight Technologies, Santa Rosa, CA, United States) [51], having the ability of generating continuous wave signals from 2 MHz to 6 GHz, was used in the measurement. In order to obtain the effective conductance of ECCs at microwave frequency, a R-band RWG probe (WR-430), as shown in the inset picture, with an aperture size of 4.30 in (109.22 mm) × 2.15 in (54.61 mm) was used as the detecting sensor which was connected to an output terminal of the handheld VNA. The specimens were naturally cured by covering a plastic sheet and had surface moisture removed prior to testing.

In this mND study, a direct measurement method in two-step was used, where the normalized impedance in complex form *z_L_*(*ω*) = *r*(*ω*) + *jx*(*ω*) was recorded in the VAN and then converted to admittance *y_L_*(*ω*) = 1/*z_L_*(*ω*) = *g*(*ω*) + *jb*(*ω*). In theory, the complex reflection coefficient (*T*) was firstly computed by the VNA via the reflected signal from the ECC specimen under test, as shown in Figure 2. The reflection coefficient with respect to the aperture of the RWG sensor holds the relationship with microwave admittance as given by [25]:

(1) $$\Gamma \left(\omega \right)=\frac{1-y_{L}\left(\omega \right)}{1+y_{L}\left(\omega \right)}=\frac{1-g\left(\omega \right)-jb\left(\omega \right)}{1+g\left(\omega \right)+jb\left(\omega \right)}$$

where *g*(*ω*) and *b*(*ω*) are, respectively, the normalized conductance and normalized susceptance at frequency *ω*, and *j* is square root of −1.

The use of electrical conductance at microwave frequency for mDNI instead of the reflection coefficient in CBMs [16,17,18,19,20] tends to be more understandable in civil construction engineering. Measurements of electrical resistance at low frequencies including direct current (DC) were used in a number of studies for structure health monitoring as well as CMBs characterization [11,12,13,14,15,52]. The effective resistance or effective conductance measurement does not involve any embedment or attachment; thus, it does not suffer from problems of sensor misalignment or heavy preparation work. Moreover, the electrical conductance is directly related to dielectric properties of CBMs at early ages, therefore well suited for measuring of early-age CBMs during the hydration process.

### 2.3. Four-Point Bending Test

**Figure 3:**
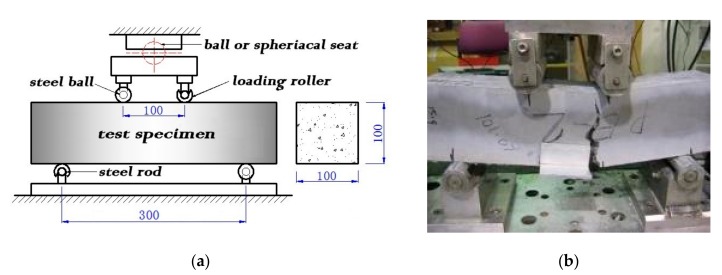
Experimental setup of four-point bending test: (**a**) schematic diagram; (**b**) photo of PE2 specimen under test.

In order to get the values of flexural strength, prism specimens with a size of 100 mm × 100 mm × 400 mm, which was cast in accordance to AS 1012.8.2, were used to perform a four-point bending test for each mixture to examine the modulus of rupture of ECC. The schematic of the four-point bending test in accordance with Australian standards AS 1012.11-2000 is illustrated in Figure 3. Before the testing, the specimens were kept wet for at least 48 h as recommended by the standard. During the test, a sealing load not exceeding the value of 100 N was applied, and the position of the contact between the loading rollers and the specimen surface was marked on the specimen side before the beginning of loading. The equivalent bending stress with a rate of 0.9–1.1 MPa/min was carefully applied without shock, and the load was increased until no further force could be sustained. The values of flexural strength in term of MOR were recorded by the PC-based machine as shown in Figure 3b.

### 2.4. Modulus of Elasticity Measurement

**Figure 4:**
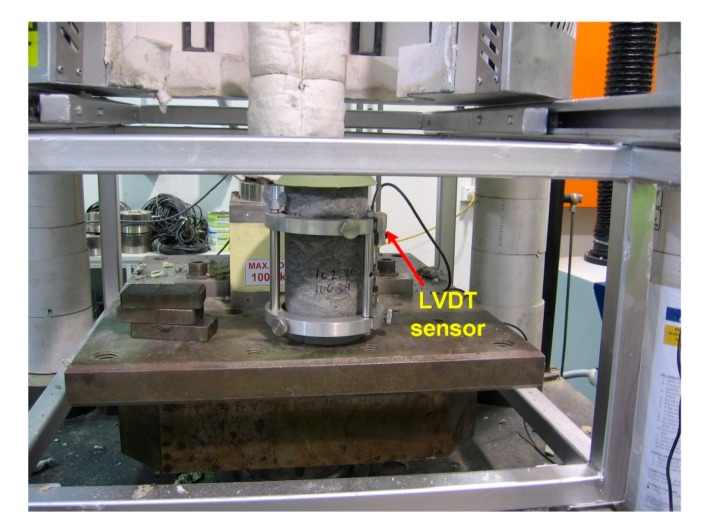
Photo of experimental fixture of modulus of elasticity test.

An accurate estimation of the modulus of elasticity is vital for proper structural design of concrete members. In particular, the time-dependent development estimation of modulus of elasticity during curing ages, especially at early age, is needed for structural health monitoring (SHM). In this study, cylindrical specimens in accordance to AS 1012.9 measuring 100 mm in diameter, 200 mm in height were prepared. The maximum period of time of the removal of specimens from the curing condition prior to the testing should not be more than 30 min. During the measurement, the applied load with a rate within the range 13–17 MPa/min was applied carefully without shock. The MOE value was obtained based on the compressive strength of ECCs, which requires a test load of 40% of the average compressive strength of cylindrical specimens according to Australian standards AS 1012.17-1997. Specimens were loaded at least three times, and readings of the first loading cycle were discarded due to the seating of gauges. It should be mentioned that all mechanical tests were undertaken inside a laboratory where a temperature of 22–26 °C and humidity of 45–55% were maintained. Figure 4 depicts the experimental setup of the MOE test where an ECC cylindrical specimen was mounted with one LVDT sensor.

## 3. Experimental Results and Mathematical Modeling

### 3.1. Modeling of Measured Microwave Properties

**Figure 5:**
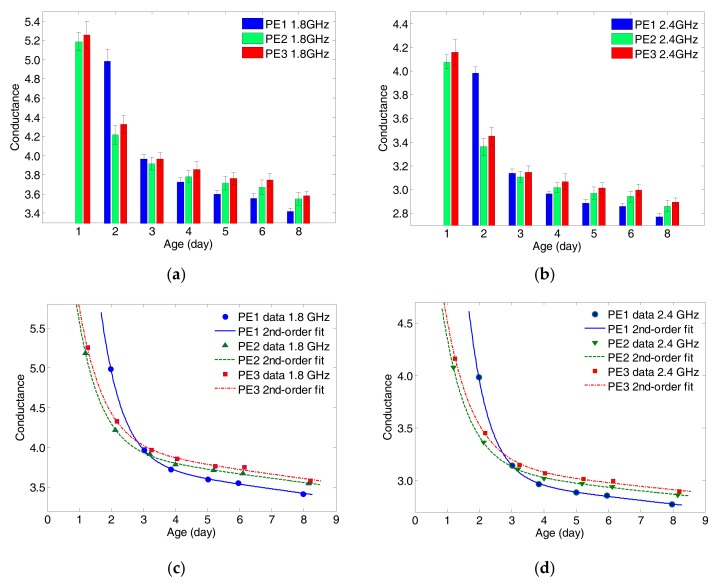
Measured average conductance with standard deviations: (**a**) 1.8 GHz; (**b**) 2.4 GHz; and modeled by using dual-frequency bi-exponential regressions: (**c**) 1.8 GHz; (**d**) 2.4 GHz.

Microwave near-field inspection technique was employed in this study, where the R-band RWG probe was used as a reflection sensor to monitor the hydration process of ECC specimens with different *w*/*b* ratios. As described in Section 2.2, the aperture of RWG probe was attached against the specimen surface to measure the microwave properties through the comparison between the incident wave and the reflected wave from the probe-specimen interface, as shown in Figure 2. In daily measurement of ECC specimens, the mNDI was undertaken for PE2 and PE3 specimens from Day-1 to Day-8, whereas measurement of PE1 commenced on Day-2 due to its late casting and hydration. For each specimen, 10 measurements were performed on non-overlapping locations of each side surface excluding the top and bottom surfaces which may have high surface roughness. A total of 40 readings of normalized electrical impedance for each specimen were recorded. The average values were used to represent the daily variations of microwave properties at the dual frequency of 1.8 GHz and 2.4 GHz. In order to achieve accurate measurement results, a calibration was of great importance at the beginning of each daily measurement [25]. Figure 5a,b depict the measured daily conductance data whereas Figure 5c,d display the average values fitted by the bi-exponential (2^nd^ order) curves as given in Equation (2).

(2a) $$g_{f1}\left(t\right)=K_{1}\;exp\left(-\frac{t}{m}\right)+K_{2}\;exp\left(-\frac{t}{n}\right),\;2\leq t\leq 8,\;f=1.8,\;2.4\;\mathrm{GHz}$$

(2b) $$g_{f2,3}\left(t\right)=K_{1}\;exp\left(-\frac{t}{m}\right)+K_{2}\;exp\left(-\frac{t}{n}\right),\;1\leq t\leq 8,\;f=1.8,\;2.4\;\mathrm{GHz}$$

where *g_f_*_1_ and *g_f_*_2,3_ are the normalized microwave conductance of PE1, PE2 & PE3 specimens, respectively, at frequency *f*; *K*_1_, *K*_2_, *m* and *n* are the regression coefficients. These values and goodness-of-fit values (R^2^) are listed in Table 5 and Table 6 for the dual frequency of 1.8 GHz and 2.4 GHz, respectively.

**Figure 6:**
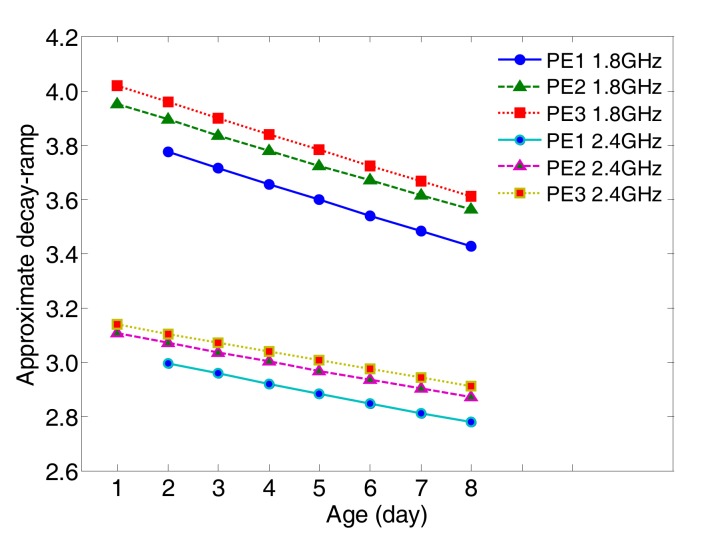
Approximate decay-ramp functions of Equation (2a,b) at 1.8 GHz and 2.4 GHz.

It can be observed from Figure 5 that the bi-exponential functions well fitted the measured data with very high R^2^ values whilst the regression curves at the two frequencies (1.8 & 2.4 GHz) have high similarities. The bi-exponential functions can be decomposed in two components: a normalized exponential decayed function and an approximate decay-ramp function. The time constants of the exponential decayed functions, which indicate the initial reducing rate of conductance, increase monotonically with increasing *w*/*b* ratios, as shown in Table 5 and Table 6. This implies logically that the PE1 specimen (lowest *w*/*b*) has the fastest hydration rate as represented by the fastest decay of conductance at microwave frequency. Moreover, the approximate decay-ramp functions of conductance are distinguishable in view of *w*/*b* ratios, as shown in Figure 6. It is noteworthy to mention that the slopes of decay-ramps at lower frequency (1.8 GHz) are higher than that at 2.4 GHz, which may provide a means of verification on the reduction of moisture (free water) inside the specimens at the early ages.

### 3.2. Modeling of Measured Modulus of Rupture

**Figure 7:**
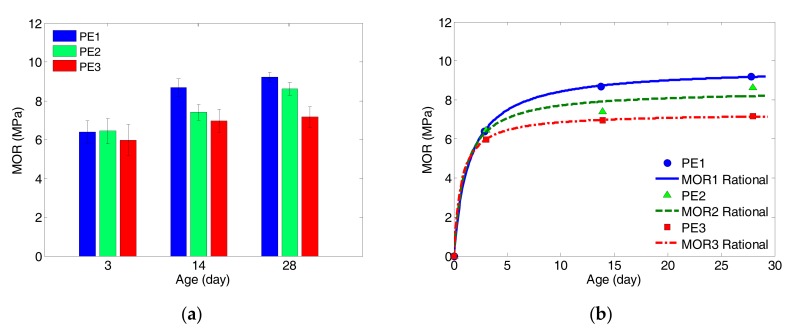
(**a**) Measured average values of MOR with standard deviations; (**b**) regression for measured data by first-order rational function.

In this study, the measured results of MOR obtained via the four-point bending test are presented, and they were modelled by using the first-order rational function as given by Equation (3). Figure 7 displays the measured data of all specimens and the corresponding regression curves, whereas Table 7 summarizes all measured MOR average values with standard deviations, regression coefficients and goodness-of-fit values. It is observed that the data were well-fitted by the proposed functions, which demonstrate the growth of MOR of ECC specimens with different *w*/*b* ratios against curing age. It is also noted that the regression coefficients (*a* and *b*) are monotonically decreasing with increasing *w*/*b* ratios. This indicates that the growth of MOR of ECC specimens can be logically represented by the regression coefficients of *a* and *b*:

(3) $$\mathrm{MOR}\left(t,\frac{w}{b}\right)=\frac{t}{\left(w/b\right)\left(a+bt\right)},\;0\leq t\leq 28$$

where *t* is the time/age variable in unit of day, *a* and *b* are the regression coefficients. The R^2^ values are all obtained higher than 0.98.

It is understandable to have the effects of *w*/*b* on the growth of MOR of ECC specimens as illustrated in Figure 7. The results indicate that the 28-day values of MOR decrease with increasing *w*/*b*, and growing with curing age, which are consistent with most experimental results of cementitious materials. This can be explained by the fact that the higher the initial water content leads to higher internal flaws such as coarse pores in matrix. This directly reduces the fracture toughness of the fiber-matrix interface, which in turn, reduces the flexural strength (MOR) of ECC [38]. Moreover, it is observed that the growing rate of MOR is far higher than the compressive strength of the same materials as reported in [30]. After 3 days of curing, the average MOR reads 69~84% of the 28-day’s values while the compressive strength reaches about 40%.

### 3.3. Modeling of Measured Modulus of Elasticity

**Figure 8:**
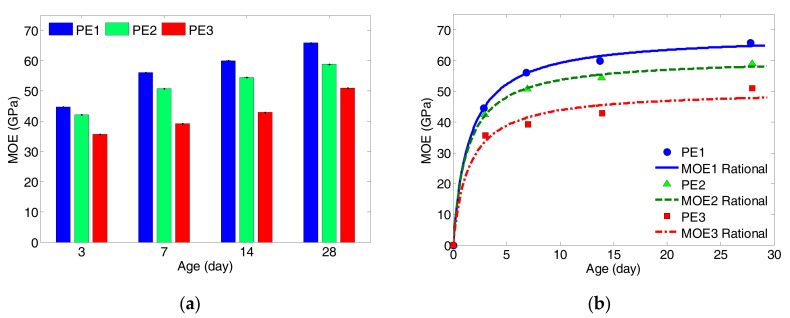
(**a**) Measured single values of MOE; (**b**) regression for measured data by first-order rational function.

The modulus of elasticity, which describes the degree of elastic of the material, is one of the most important mechanical properties of the ductile CBMs. The significance of the MOE is usually reflected in the stiffness of the materials, which is interrelated with flexural strength and compressive strength. A better understanding of elastic behavior of ECC is crucial for its applications in structural engineering. In order to obtain the modulus of elasticity of ECC specimens, a test load of 40 percent of the average compressive strength was applied to the molded cylinders in this study. By observing the growing data of MOE, the measured results were modelled by using the first-order rational regression as a function of *w*/*b* ratio, as given by:

(4) $$\mathrm{MOE}\left(t,\frac{w}{b}\right)=\frac{t}{\left(w/b\right)\left(c+dt\right)},\;0\leq t\leq 28$$

where *t* is the time/age variable in unit of day, *c* and *d* are the regression coefficients. All the measurement data, regression coefficients and the goodness-of-fit values are listed in Table 8, whereas the regression curves are plotted against the curing age as presented in Figure 8.

It is observed from Figure 8 that the MOE values of ECC specimens grow exponentially with the curing age. The values of MOR rapidly increase during the first 7 days, particularly for the specimens with the lowest *w*/*b* ratio (PE1). After 7 days, the rate of growing remains as a slow increase until 28 days. Moreover, it has been clearly observed that there is a trend of increasing values of MOE with decreasing *w*/*b* ratio. The growth of MOE can be explained based on Power’s Model [45], as the hydration degree increases and the hydration products grow, the volume of water reduces. As a result, the MOE increases with the curing age. Although the differences in *w*/*b* ratio are small, they have a great influence on the volume of remaining water during hydration process. It is worth noting that, although only single cylindrical specimen per type was used in the MOE test, the overall results and modelling are found to be logical and reasonable.

## 4. Correlation and Prediction Models

### 4.1. Correlation between MOR and Conductance

**Figure 9:**
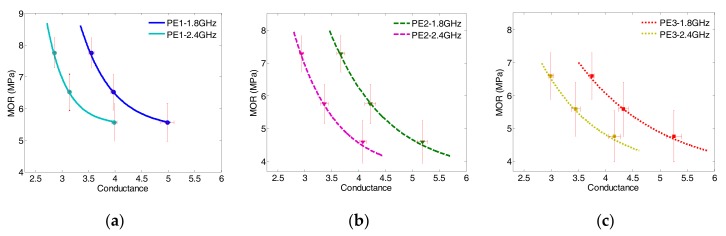
MOR-Conductance correlation using dual frequency for (**a**) PE1 specimen; (**b**) PE2 specimen; (**c**) PE3 specimen.

In this section, the correlation between MOR and normalized conductance at dual frequency is taken into consideration by mapping Equations (2) and (3) based on the regression models of the measurement data described in previous sections. The variation of conductance at 1.8 GHz and 2.4 GHz is used for comparison. The purposes are to obtain the correlation model in the early age of first 8 days using dual frequency and hence fulfilling the prediction purpose. Equation (5) describes the exponential correlations of MOR with conductance (*g*) of all ECC specimens at the frequencies of 1.8 GHz and 2.4 GHz. Figure 9 shows the comparison of proposed models for various ECC specimens where the performances were verified by the experimental data:

(5) $$\mathrm{MOR}\left(g_{f},\frac{w}{b}\right)=A_{f}exp\left(-\frac{g_{f}}{m_{f}}\right)+C_{f}\left(\frac{w}{b}\right),\;f=1.8,\;2.4\;\mathrm{GHz}$$

where $g_{f}$ is the normalized conductance at the dual frequency of 1.8 GHz and 2.4 GHz, and *A_f_*, *C_f_*, and $m_{f}$ are the regression coefficients at the corresponding frequency. The regression coefficients and goodness of fits for Equation (5) are summarized in Table 9 and Table 10, respectively.

As seen from Figure 9, it is clearly observed that the moduli of rupture of all ECC specimens are monotonically decreasing with the increasing conductance. The value of MOR of a particular ECC specimen varies depending on frequency. It can also be observed that the trend of each type of composites at different frequency is similar, as the tangents of each specimen type of curves at dual frequency are virtually in parallel. For a given conductance, the values of MOR of all ECCs mapped at 1.8 GHz are higher than that read from 2.4 GHz curves. Moreover, the decay range of each ECC specimen is the same at the two frequencies. Therefore, one can use the different conductance values at dual frequency to verify the predicted MOR value. This shows the advantage when using dual frequency correlation over the single frequency one.

**Figure 10:**
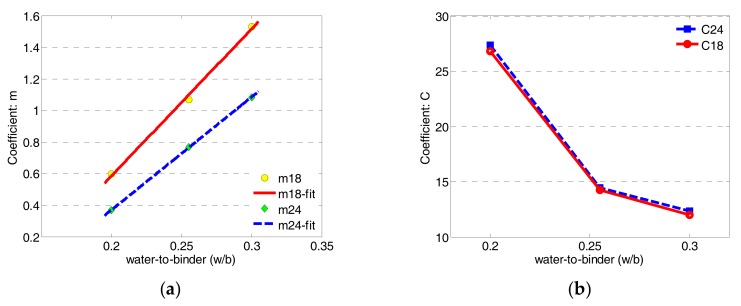
Comparison of regression coefficients versus *w*/*b* at dual frequency: (**a**) regression coefficient *m*; (**b**) regression coefficient *C*.

For a better understanding of the modelling correlation between MOR and conductance using first-order exponential regression, the regression coefficients of *m* and *C* versus *w*/*b* at the dual frequency are plotted as shown in Figure 10. It is observed from Figure 10a that the coefficient *m* is varying linearly with increasing *w*/*b*, whereas the increasing slope at 1.8 GHz is higher than that at 2.4 GHz. This feature can be used as an indication for distinguishing the ECC specimens with different initial water contents. Meanwhile, Figure 10b indicates the variations of coefficient *C* at the dual frequency versus *w*/*b*, which are almost overlapped. These imply that the final values of MOR (*g*) are the same although the values of conductance at the dual frequency are different. Therefore, this dual-frequency correlation can be used for structural health monitoring, which is not just for prediction purpose but also for verification of the predication of mechanical properties of ECCs.

### 4.2. Correlation Model between MOE and Conductance

In the same manner of MOR, by data mapping Equations (2) and (4), the correlation between MOE and the microwave conductance of ECC specimens can be obtained, via the common variable of early age within the first 8 days, as given by:

(6) $$\mathrm{MOE}\left(g_{f},\frac{w}{b}\right)=G_{f}exp\left(-\frac{g_{f}}{k_{f}}\right)+H_{f}\left(\frac{w}{b}\right),\;f=1.8,\;2.4\;\mathrm{GHz}$$

where $g_{f}$ is the normalized conductance at the dual frequency of 1.8 GHz and 2.4 GHz, and *G_f_*, *H_f_*, and $k_{f}$ are the regression coefficients at the corresponding frequency. The regression coefficients and goodness of fits for Equations (6) are summarized in Table 11 and Table 12, respectively.

**Figure 11:**
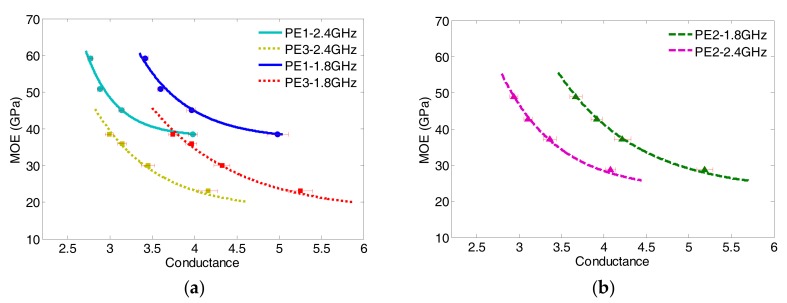
MOE-Conductance correlation using dual frequency for (**a**) comparison of PE1 and PE3 specimens; (**b**) only PE2 specimen.

Figure 11 shows the performance of the correlation of each ECC specimens at dual frequency, and verified with measurement data. It is observed that, similar to the MOR scenarios, the modulus of elasticity is monotonically decreasing with the increasing conductance whereas the slopes of the dual-frequency correlation models/curves are virtually in parallel with each other. The final values (*H_f_*) and the decay constants (*k_f_*) obtained as shown in Table 11 and Table 12 are consistence with that obtained from the MOR cases. By comparing the performance of correlation models of MOR (Figure 9) and MOE (Figure 11), one can observe that the MOE variations are more sensitive to the *w*/*b* ratio than the MORs under the same conditions.

### 4.3. Relation between MOE and MOR

It is interesting to know the relation between the measured MOE and measured MOR data as it provides a means of estimation for the other quantities when one of the mechanical properties is known. The measured data of MOE correlated with the measured MOR for the 7th, 14th and 28th day fitted by a linear function is given by:

(7) MOE=8.32×MOR−11.18, 6≤MOR≤10 MPa

**Figure 12:**
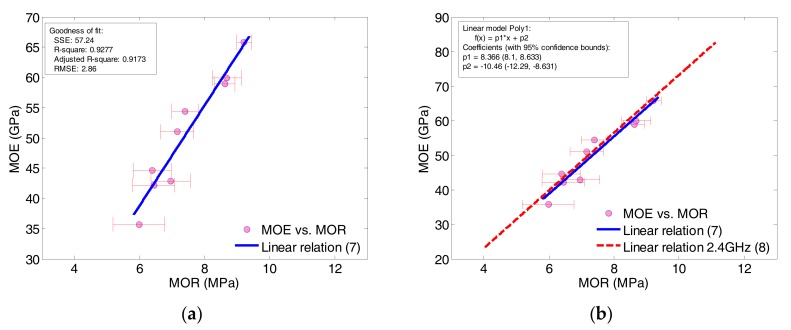
Relations between MOE in GPa and MOR in MPa of ECC specimens: (**a**) obtained by fitting measured data; (**b**) verified by mapping Equations (5) and (6).

The linear relation obtained is based on all measured data on the abovementioned days regardless of *w*/*b* ratio, as shown in Figure 12a. The linear function (7) has a goodness-of-fit value of R^2^ = 0.928 with the measured data presented. At the mid-range of 8 MPa, the MOE value reads 55.38 GPa from Equation (7). It should be mentioned that this linear relation is valid only for the ECC specimens with *w*/*b* range of 0.20–0.30 as described per Section 2. As a matter of cross-verification, the relation between MOR and MOE can also be obtained by mapping Equations (5) and (6) by substituting proper values of conductance at 1.8 or 2.4 GHz. A linear relation at 2.4 GHz is then obtained and compared with the experimental one (7), as shown in Figure 12b, where the linear relation with an extended range of MOR is given by:

(8) MOE=8.37×MOR−10.46, 4≤MOR≤12 MPa

At the mid-range of 8 MPa, the new linear relation (8) gives a MOE value of 56.5 GPa, which in turn gives an error of +2%. From Figure 12b, it is observed that the two linear relations of MOE vs. MOR are virtually coincident with each other.

## 5. Conclusions

This article presents the prediction of strength and elasticity of engineered cementitious composites in terms of modulus of rupture (MOR) and modulus of elasticity (MOE), respectively, by utilizing the microwave nondestructive technique. The investigation was conducted in the sense of cross-disciplinary experimental programs. The developed correlation models, however, are only specific to the cast and tested ECC specimens described. If generalized model(s) are expected to be developed, extended experimental data is required and needs to be obtained from a large number of specimens with different mix proportions and PVA fiber volume ratios. In this study, the dual-frequency correlation models of MOR and MOE against effective conductance were developed. The following findings were summarized within the scope of this study:

1. It was found that the first-order rational function is well-suited for the growth modeling of MOR and MOE of ECC specimens as a function of curing age and *w*/*b* ratio.
2. The growing rates and the mature values of MOR and MOE are decreasing with the increasing *w*/*b*, which can be effectively inspected and predicted by using microwave conductance.
3. The achieved correction models indicate that both the MOR and MOE of ECC specimens developed as a function of conductance at dual frequency, which can be used for structural health monitoring, not just for prediction but also for a verification purpose. This is the key advantage of using dual-frequency correlations.
4. The linear relation between MOR and MOE is obtained by their predicting functions, which demonstrate the reliability of the microwave technique in monitoring comparing with the measured data.
5. It is feasible to monitor and predict the working performance of ECC or CBMs in on-site engineering projects by using handheld VNA via means of microwave nondestructive technique.

## Figures and Tables

**Table 1:** Mechanical and physical properties of PVA fiber.

Fiber	Tensile Strength (MPa)	Elastic Modulus (GPa)	Specific Gravity	Diameter (μm)	Length (mm)	Shape	Color
PVA	1600	40	1.3	38	8	straight	white

**Table 2:** Chemical composition of Portland cement (PC), Fine sand (FS) and Fly ash (FA-F).

Constituent	FS (% wt)	PC (% wt)	FA-F (% wt)
CaO	-	58.1	-
SiO_2_	95.6	24.7	76.3
Al_2_O_3_	1.9	6.4	20.2
SO_3_	0.3	3.5	-
MgO	-	2.4	-
FeO	0.3	2.2	0.4
TiO_2_	0.9	-	0.9
K_2_O	-	0.5	0.4
LOI	1.0	2.2	1.8

**Table 3:** Mix proportions of ECC composites (kg/m^3^).

ECC	*w*/*b*	Cement	Fly Ash	Water	Sand	HRWR	PVA (%vol)
PE1	0.200	618	742	272	494	9.83	2
PE2	0.255	578	694	324	462	9.19	2
PE3	0.300	544	654	359	435	8.66	2

**Table 4:** Various dimensions of cast ECC specimens.

Test	Standard	Shape	Sizes (mm)	No. of Test Specimens/Type
Microwave near-field Test	custom	cube	200 × 200 × 200	1
Four-point bending Test	AS1012.8.2	rectangular prism	100 × 100 × 400	3
Modulus of elasticity Test	AS 1012.9	cylinder	Ø100, H200	1

**Table 5:** Regression coefficients and goodness of fits for measured data at 1.8 GHz.

1.8 GHz	*w*/*b*	*K*_1_	*m*	*K*_2_	*n*	R^2^
PE1	0.200	24.17	0.664	3.90	61.73	0.9998
PE2	0.255	6.65	0.718	4.01	67.55	0.9995
PE3	0.300	6.39	0.773	4.08	65.64	0.9984

**Table 6:** Regression coefficients and goodness of fits for measured data at 2.4 GHz.

2.4 GHz	*w*/*b*	*K*_1_	*m*	*K*_2_	*n*	R^2^
PE1	0.200	24.57	0.619	3.07	79.73	0.9999
PE2	0.255	4.54	0.781	3.14	88.42	0.9998
PE3	0.300	4.58	0.843	3.17	93.43	0.9991

**Table 7:** Average MOR with standard deviations, regression coefficients and goodness of fits for Equation (3).

ECC	MORav (Day-3)	MORstd (Day-3)	MORav (Day-14)	MORstd (Day-14)	MORav (Day-28)	MORstd (Day-28)	*a*	*b*	R^2^
PE1	6.39	0.58	8.68	0.45	9.22	0.23	0.756	0.518	0.9999
PE2	6.44	0.65	7.40	0.40	8.62	0.32	0.468	0.462	0.9898
PE3	5.98	0.80	6.96	0.60	7.16	0.52	0.308	0.456	1.000

**Table 8:** Measured MOE (GPa), regression coefficients and goodness of fits for Equation (4).

ECC	MOE (Day-3)	MOE (Day-7)	MOE (Day-14)	MOE (Day-28)	*c*	*d*	R^2^
PE1	44.6	56.0	59.9	65.8	0.113	0.073	0.9987
PE2	42.2	50.8	54.4	58.9	0.086	0.065	0.9991
PE3	35.7	39.2	42.9	51.0	0.099	0.066	0.9831

**Table 9:** Regression coefficients at *f* = 1.8 GHz and goodness of fits for Equation (5).

ECC	*w*/*b*	*A*_18_	*C*_18_	*m*_18_	R^2^
PE1	0.200	880.9	26.8	0.598	0.998
PE2	0.255	111.2	14.25	1.068	0.998
PE3	0.300	33.62	11.96	1.532	0.999

**Table 10:** Regression coefficients at *f* = 2.4 GHz and goodness of fits for Equation (5).

ECC	*w*/*b*	*A*_24_	*C*_24_	*m*_24_	R^2^
PE1	0.200	5238	27.38	0.367	0.998
PE2	0.255	162	14.43	0.768	0.998
PE3	0.300	44.67	12.32	1.082	0.997

**Table 11:** Regression coefficients at *f* = 1.8 GHz and goodness of fits for Equation (6).

ECC	*w*/*b*	*G*_18_	*H*_18_	*k_18_*	R^2^
PE1	0.200	6960	185.4	0.589	0.9982
PE2	0.255	1310	88.69	0.940	0.9982
PE3	0.300	905.8	57.4	1.013	0.9984

**Table 12:** Regression coefficients at *f* = 2.4 GHz and goodness of fits for Equation (6).

ECC	*w*/*b*	*G*_24_	*H*_24_	*k_24_*	R^2^
PE1	0.200	42170	189.5	0.362	0.9979
PE2	0.255	2052	90.08	0.673	0.9979
PE3	0.300	1466	59.58	0.711	0.9969

## References

[1] International Atomic Energy Agency (IAEA) (2002). Guidebook on Non-Destructive Testing of Concrete Structures.

[2] Gu H., Song G.B., Dhonde H., Mo Y.L., Yan S. (2006). Concrete early-age strength monitoring using embedded piezoelectric transducers. Smart Mater. Struct..

[3] Kim J., Lee C., Park S. (2017). Artificial neural network-based early-age concrete strength monitoring using dynamic response signals. Sensors.

[4] Qin L., Li Z. (2008). Monitoring of cement hydration using embedded piezoelectric transducers. Smart Mater. Struct..

[5] Kim J., Kim J.W., Lee C., Park S. (2017). Development of embedded EM sensors for estimating tensile forces of PSC girder bridges. Sensors.

[6] Wu T.T., Lin T.F. (1998). The stress effect on the ultrasonic velocity variations of concrete under repeated loading. Mater. J..

[7] Selleck S.F., Landis E.N., Peterson M.L., Shah S.P., Achenbach J.D. (1998). Ultrasonic investigation of concrete with distributed damage. Mater. J..

[8] Lafhaj Z., Goueygou M., Djerbi A., Kaczmarek M. (2006). Correlation between porosity, permeability and ultrasonic parameters of mortar with variable water/cement ratio and water content. Cem. Concr. Res..

[9] Yusuf I.T., Jimoh Y.A., Salami W.A. (2016). An appropriate relationship between flexural strength and compressive strength of palm kernel shell concrete. Alex. Eng. J..

[10] Hoła J., Schabowicz K. (2010). State-of-the-art non-destructive methods for diagnostic testing of building structures-anticipated development trends. Arch. Civ. Mech. Eng..

[11] Lencioni J.W., Lima M.G.D., Güneş O., Akkaya Y. (2013). A study of the parameters that affect the measurements of superficial electrical resistivity of concrete. Nondestructive Testing of Materials and Structures.

[12] Lataste J.F., Sirieix C., Breysse D., Frappa M. (2003). Electrical resistivity measurement applied to cracking assessment on reinforced concrete structures in civil engineering. NDT E Intern..

[13] Ferreira R.M., Jalali S. (2010). NDT measurements for the prediction of 28-day compressive strength. NDT E Intern..

[14] Xiao L., Li Z. (2008). Early-age hydration of fresh concrete monitored by non-contact electrical resistivity measurement. Cem. Concr. Res..

[15] Wei X., Xiao L., Li Z. (2012). Prediction of standard compressive strength of cement by the electrical resistivity measurement. Constr. Build. Mater..

[16] Zoughi R., Nowak P.S. (1999). Strength-Related Testing of Concrete Using Microwave Signals. U.S. Patent.

[17] Zoughi R., Cone G.L., Nowak P.S. (1991). Microwave non-destructive detection of rebars in concrete slabs. Mater. Eval..

[18] Bois K., Benally A., Nowak P., Zoughi R. (1998). Cure-state monitoring and water-to-cement ratio determination of fresh Portland cement-based materials using near-field microwave techniques. IEEE Trans. Instrum. Meas..

[19] Mubarak K., Bois K.J., Zoughi R. (2001). A simple, robust, and on-site microwave technique for determining water-to-cement ratio of fresh Portland cement-based materials. IEEE Trans. Instrum. Meas..

[20] Bois K., Benally A., Zoughi R. (2000). Microwave near-field reflection property analysis of concrete for material content determination. IEEE Trans. Instrum. Meas..

[21] Al-Mattarneh H.M.A., Ghodgaonkar D.K., Majid M.B.W.A.W. (2001). Microwave sensing of moisture content in concrete using open-ended rectangular waveguide. Surf. Sens. Technol. Appl..

[22] Peer S., Case J.T., Gallaher E., Kurtis K.E., Zoughi R. (2003). Microwave reflection and dielectric properties of mortar subjected to compression force and cyclically exposed to water and sodium chloride solution. IEEE Trans. Instrum. Meas..

[23] Hasar U.C. (2009). Non-destructive testing of hardened cement specimens at microwave frequencies using a simple free-space method. NDT E Int..

[24] Jamil M., Hassan M.K., Al-Mattarneh H.M.A., Zain M.F.M. (2013). Concrete dielectric properties investigation using microwave nondestructive techniques. Mater. Struct..

[25] Chung K.L., Kharkovsky S. (2015). Monitoring of microwave properties of early-age concrete and mortar specimens. IEEE Trans. Instrum. Meas..

[26] Castro A.F., Valcuende M., Vidal B. (2015). Using microwave near-field reflection measurements as a non-destructive test to determine water penetration depth of concrete. NDT E Int..

[27] Hashemi A., Horst M., Kurtis K.E., Donnell K.M., Zoughi R. (2015). Comparison of alkali–silica reaction gel behavior in mortar at microwave frequencies. IEEE Trans. Instrum. Meas..

[28] Kim S., Kang J., Lee S.H., Ahn Y.H. (2016). Effect of chlorides on conductivity and dielectric constant in hardened cement mortar: NDT for durability evaluation. Adv. Mater. Sci. Eng..

[29] Shen P., Lu L., He Y., Wang F., Hu S. (2016). Hydration monitoring and strength prediction of cement-based materials based on the dielectric properties. Constr. Build. Mater..

[30] Chung K.L., Luo J., Yuan L., Zhang C., Qu C. (2017). Strength correlation and prediction of engineered cementitious composites with microwave properties. Appl. Sci..

[31] Neville A.M. (1996). Properties of Concrete.

[32] Gonen T., Yazicioglu S. (2007). The influence of compaction pores on sorptivity and carbonation of concrete. Constr. Build. Mater..

[33] Li V.C., Wu C., Wang S., Saito T. (2002). Interface tailoring for strain-hardening PVA-ECC. ACI Mater. J..

[34] Li V.C. (2012). Tailoring ECC for special attributes: A review. J. Concr. Struct. Mater..

[35] Li V.C., Nawy E. (2008). Engineered cementitious composites (ECC)-material, structural, and durability performance. Concrete Construction Engineering Handbook.

[36] Ma H., Qian S., Zhang Z. (2014). Effect of self-healing on water permeability and mechanical property of medium-early-strength engineered cementitious composites. Constr. Build. Mater..

[37] Chung K.L., Ghannam M., Zhang C. (2017). Effect of specimen shapes on compressive strength of engineered cementitious composites (ECCs) with different values of water-to-binder ratio and PVA fiber. Arab. J. Sci. Eng..

[38] Yang Y., Gao X., Deng H., Yu P., Yao Y. (2010). Effects of water/binder ratio on the properties of engineered cementitious composites. J. Wuhan Univ. Technol.-Mater. Sci. Ed..

[39] Cong L., Leung C.K.Y. (2017). Theoretical evaluation of fiber orientation and its effects on mechanical properties in engineered cementitious composites (ECC) with various thicknesses. Cem. Concr. Res..

[40] Lu C., Leung C.K.Y., Li V.C. (2017). Numerical model on the stress field and multiple cracking behavior of engineered cementitious composites (ECC). Constr. Build. Mater..

[41] Zhou J., Qian S., Ye G., Copuroglu O., Breugel K.V., Li V.C. (2012). Improved fiber distribution and mechanical properties of engineered cementitious composites by adjusting the mixing sequence. Cem. Concr. Compos..

[42] Soe K.T., Zhang Y.X., Zhang L.C. (2013). Impact resistance of hybrid-fiber engineered cementitious composite panels. Compos. Struct..

[43] Nurdeen M.A., Johari M.A.M., Hashim S.F.S. (2012). Flexural performance of green engineered cementitious composites containing high volume of palm oil fuel ash. Constr. Build. Mater..

[44] Yildirim G., Keskin O.K., Keskin S.B., Sahmaran M., Lachemi M. (2015). A review of intrinsic self-healing capability of engineered cementitious composites: Recovery of transport and mechanical properties. Constr. Build. Mater..

[45] Martínez-Molina W., Torres-Acosta A.A., Jáuregui J.C., Chávez-García H.L., Alonso-Guzmán M., Graff E.M., Arteaga-Arcos J.C. (2014). Predicting concrete compressive strength and modulus of rupture using different NDT techniques. Adv. Mater. Sci. Eng..

[46] Yoshitake I., Rajabipour F., Yoichi M., Andrew S. (2012). A prediction method of tensile Young’s modulus of concrete at early age. Adv. Civ. Eng..

[47] Tian Z., Bian C. (2013). Numerical modeling of elastic modulus for cement paste using homogenization method. J. Wuhan Univ. Technol.-Mater. Sci. Ed..

[48] Wu L., Yan B., Lei B. (2013). Prediction of elastic modulus of cement-based materials based on power’s volume model. Appl. Mech. Mater..

[49] Yapici F., Ozcifci A., Akbulut T., Bayir R. (2009). Determination of modulus of rupture and modulus of elasticity on flakeboard with fuzzy logic classifier. Mater. Des..

[50] Tiryaki S., Hamzacebi C. (2014). Predicting modulus of rupture (MOR) and modulus of elasticity (MOE) of heat treated woods by artificial neural networks. Measurement.

[51] Keysight Technologies (2016). N9923A FieldFox RF Vector Network Analyzer Data Sheet.

[52] Chung D.D.L. (2001). Structural health monitoring by electrical resistance measurement. Smart Mater. Struct..

